# Atomistic Origin of Photoluminescence Quenching in Colloidal MoS_2_ and WS_2_ Nanoplatelets

**DOI:** 10.1021/acs.nanolett.5c05893

**Published:** 2026-02-28

**Authors:** Surender Kumar, Markus Fröhlich, Stefan Velja, Marco Kögel, Onno Strolka, André Niebur, Samuell Ginzburg, Muhammad Sufyan Ramzan, Jannik C. Meyer, Jannika Lauth, Caterina Cocchi

**Affiliations:** † Institut für Festkörpertheorie und -Optik, 9378 Friedrich-Schiller-Universität Jena, 07743 Jena, Germany; ‡ Institute of Physical and Theoretical Chemistry, 9188 Eberhard Karls University of Tübingen, 72076 Tübingen, Germany; ¶ 232751 NMI Natural and Medical Sciences Institute at the University of Tübingen, 72770 Reutlingen, Germany; § 200992 University of Tübingen, Institute of Applied Physics, 72076 Tübingen, Germany; ∥ 234487 Leibniz University of Hannover, Cluster of Excellence PhoenixD (Photonics, Optics and Engineering - Innovation Across Dimensions), 30167 Hannover, Germany; ⊥ Yusuf Hamied Department of Chemistry, 26555 University of Cambridge, Lensfield Road, CB2 1EW Cambridge, U.K.

**Keywords:** transient absorption spectroscopy, photoluminescence quenching, DFT, edge states, MoS_2_ and WS_2_ nanoplatelets

## Abstract

Large chemical tunability and strong light–matter interactions make colloidal transition metal dichalcogenide (TMD) nanostructures particularly suitable for light-emitting applications. However, ultrafast exciton decay and quenched photoluminescence (PL) limit their potential. Combining femtosecond transient absorption spectroscopy with first-principles calculations on MoS_2_ and WS_2_ nanoplatelets, we reveal that the observed sub-picosecond exciton decay originates from edge-located optically bright hole traps. These intrinsic trap states stem from the metal *d*-orbitals and persist even when the sulfur-terminated edges are hydrogen-passivated. Notably, WS_2_ nanostructures show more localized and optically active edge states than their MoS_2_ counterparts, and zigzag edges exhibit a higher trap density than armchair edges. The nanoplatelet size dictates the competition between ultrafast edge-trapping and slower core–exciton recombination, and the states responsible for exciton quenching enhance the catalytic activity. Our work represents an important step forward in understanding exciton quenching in TMD nanoplatelets and stimulates additional research to refine physicochemical protocols for enhanced PL.

## Graphical abstract

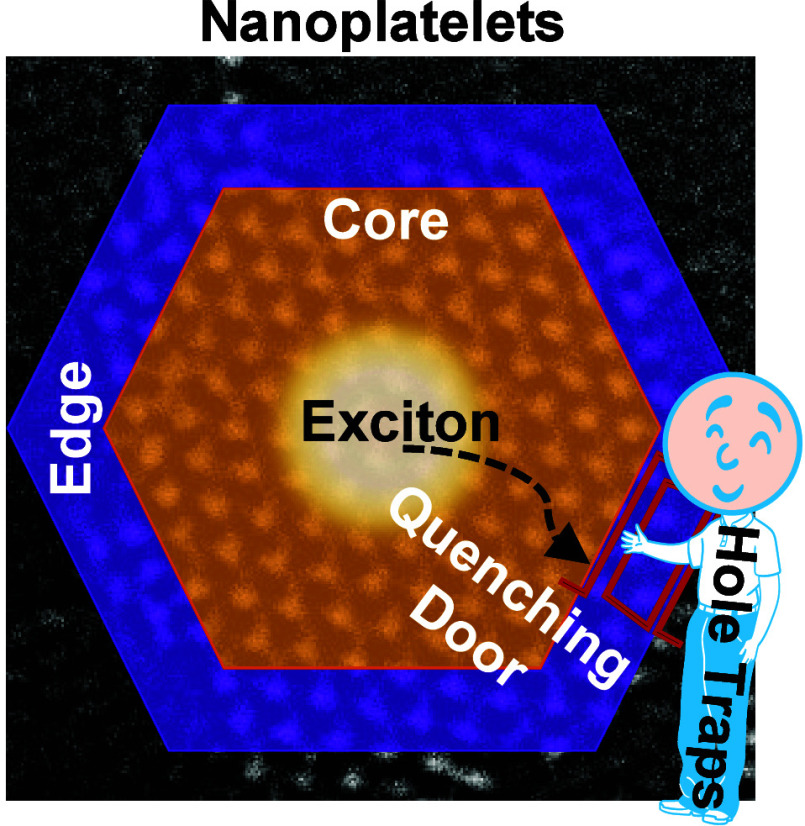

Substrate-free colloidal synthesis is a scalable and versatile bottom-up method to produce transition metal dichalcogenide (TMD) nanostructures.^1–6^ This approach offers thermodynamically stable 2H-phase monolayers^5^ with lateral sizes down to a few nanometers,^4,5,7–9^ enabling fine control over composition and surface chemistry.^1–5,8,9^ Among low-dimensional TMDs, MoS_2_ and WS_2_ stand out for their direct visible-range bandgaps, strong light–matter coupling, and tunable optical properties,^5,10,11,11–22^ which make them highly promising for thin-film integration in optoelectronic devices.

Bright photoluminescence (PL), long-lived emissive states, and tunable emission spectra are highly desirable properties for light-emitting applications. TMDs exhibit these optimal optical features thanks to their exceptionally bound excitons (∼0.5 eV binding energies).^21,23^ Although excitons are expected to radiatively recombine with high quantum efficiency,^24–27^ colloidal TMD nanostructures typically show weak or quenched PL^28–32^ compared to monolayers obtained via exfoliation/chemical vapor deposition (CVD).^33–38^ This PL loss can originate from nonradiative recombination at surface or edge sites,^10–13^ charge carrier trapping,^14,28,30,31,33,39,40^ and insufficient chemical passivation of surface states.^15,16,41^ However, the intrinsically disordered nature of colloidal samples makes it nontrivial to isolate the specific physical contributions. As a result, the fundamental atomistic origin of PL quenching and charge carrier trapping in colloidal TMD nanostructures remains unclear to date.

*Ab initio* structure calculations represent a valuable resource to complement the structural analysis and spectroscopic characterization of colloidal systems. Density functional theory (DFT) has been successfully applied to determine trap states in III–V and II–VI colloidal nanostructures,^42–46^ revealing their microscopic origin and electronic character. These exemplary results demonstrate the potential of this parameter-free approach, which can contribute to accurately identifying the microscopic origin of PL quenching in colloidal TMD nanostructures by systematically investigating different compositions, sizes, and terminations with atomistic control.

By combining transient absorption (TA) spectroscopy with DFT calculations, we identify edge-localized metal *d*-orbitals acting as trap states to be responsible for ultrafast exciton decay in colloidal WS_2_ and MoS_2_ nanoplatelets (NPLs), driving sub-picosecond PL quenching. Furthermore, we reveal how the optical properties of TMD NPLs evolve with size and how their edge states, while detrimental to PL, are key to their catalytic activity. Our findings establish a clear link between material morphology, fundamental photophysics, and functional performance, providing a rational basis for the design of versatile wet-chemically synthesized TMD nanostructures.

MoS_2_ and WS_2_ colloidal NPLs and nanosheets (NSs) [Figure 1] are synthesized by dropwise addition of MoCl_5_/WCl_6_ dissolved in oleylamine (OlAm) into a 320 °C hot solution of elemental sulfur and hexamethyldisilazane (HMDS) in OlAm.^1^ A short addition time of 10 min and using sulfur in excess (∼700 equiv) limits the lateral growth, resulting in NPLs.^7^ A longer addition time and a lower sulfur excess (3.4 equiv) on the other hand yield laterally larger NSs.^6–8,47^ Due to agglomeration caused by solvent evaporation, the NPLs arrange in macroscopic structures, resulting in the observed moiré patterns. OlAm ligands used in the synthesis ensure the spatial separation of the NPLs/NSs even when agglomerated and have been reported previously.^8,32,48^ MoS_2_ NPLs have an average lateral size of 3.7 ± 1.6 nm, and NSs have a size of 19.7 ± 4.1 nm [Figure 1(c)]. WS_2_ NPLs and NSs [Figure 1(d)] show similar sizes (3.7 ± 1.7 nm and 21.2 ± 5.0 nm). Both NPLs and NSs have a hexagonal 2H-phase crystal structure, highlighted in the fast Fourier transform (FFT) inset of Figure 1(a,b).

**Figure 1:**
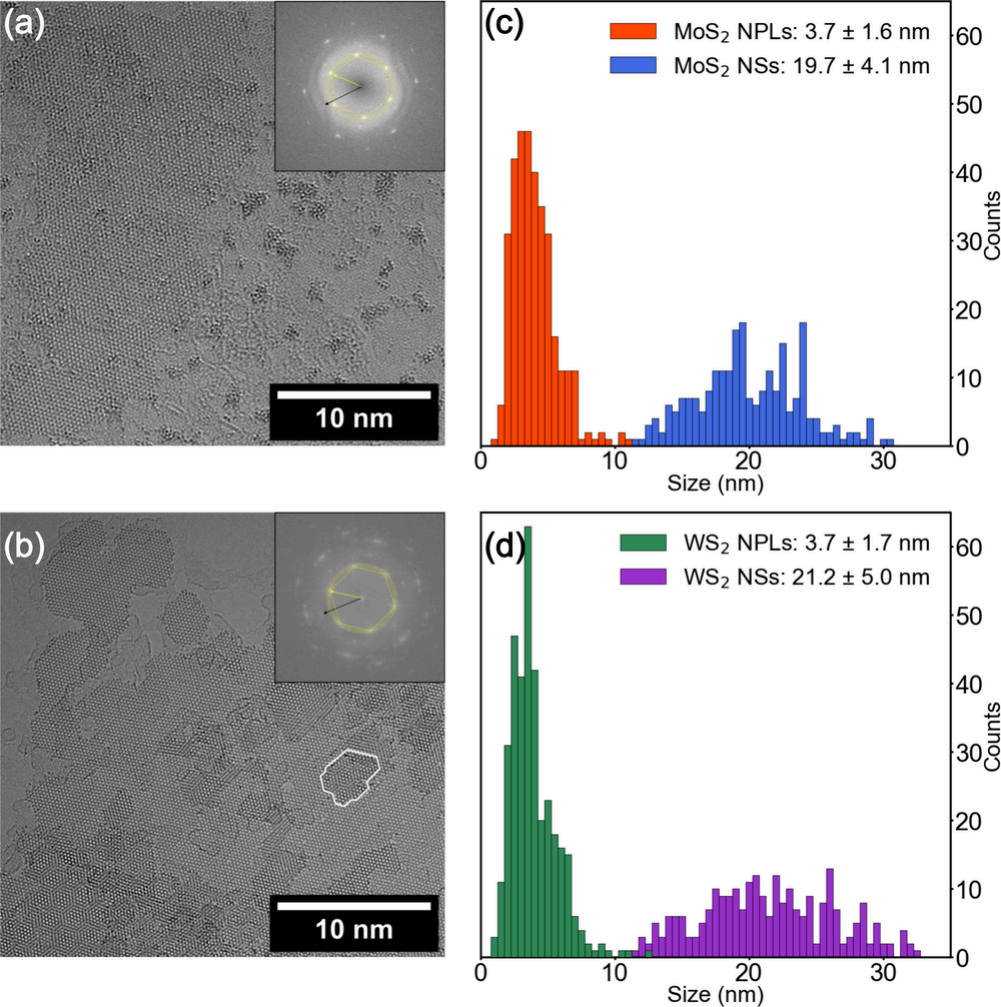
High-resolution TEM image of (a) MoS_2_ NSs and (b) WS_2_ NPLs, different in shape, size, and orientation. A single exemplary NPL is highlighted with a white border. The hexagonal crystal structure of the semiconducting 2H-phase can be seen in the FFT inset (yellow) with the underlying graphene structure indicated by the black arrow. Size distributions of (c) MoS_2_ and (d) WS_2_ NPLs and NSs, respectively.

The semiconducting 2H-phase purity and monolayer nature of these TMDs have been confirmed in previous studies by means of X-ray photoelectron spectroscopy, X-ray diffraction, and Raman spectroscopy, showing no evidence of metallic polytypes.^7,8^ Our established annealing protocol ensures a quantitative transition to the 2H-phase even in high-surface-area NPLs.^7^ The monolayer character of the samples is further corroborated by excitonic peak positions and the observation of second-harmonic generation.^32^ While individual atomic force microscope thickness measurements are complicated by the propensity of these NPLs to form ligand-separated “nanoflower” aggregates upon drying, the ensemble structural and optical fingerprints are consistent with high-quality monolayers.^32^

Compared to the larger NSs, the reduced size of NPLs introduces significant lateral confinement,^28^ resulting in a hypsochromic shift of the excitonic resonances. In MoS_2_ the A/B excitons shift from 1.91/2.06 eV (649/601 nm) in NSs to 2.04/2.18 eV (609/568 nm) in NPLs. In contrast, the shifts in WS_2_ are 2.02/2.39 eV (614/518 nm) in NSs and 2.04/2.45 eV (607/506 nm) in NPLs (see Figure S1 and Table S1). Notably, the A exciton of MoS_2_ exhibits a much larger shift (130 meV, 40 nm) compared to WS_2_ (20 meV, 7 nm) while also showing a more pronounced decrease in intensity. This indicates that the same lateral confinement (from NSs to NPLs) more prominently affects the electronic structure of MoS_2_. Similarly, in earlier work, we discussed the vanishing absorption of the A excitonic transition in colloidal MoS_2_ NPLs, showing that the dominant feature at 2.09 eV (593 nm) originates from the B-exciton, shifted from 2.03 eV (610 nm) due to lateral confinement.^7,28^ The broad absorption is attributed to a combination of inhomogeneous size broadening and morphological variations [Figure 1(a,b)], since both the relative size distribution and the edge-to-core atom ratio are higher in NPLs than in the NSs. The reduced absorption intensity in NPLs is a result of the smaller NPLs and is a direct consequence of reduced oscillator strength, which scales with the lateral surface dimensions.^49–51^

The TA spectra, acquired immediately after photoexcitation (*t*_0_) [Figure 2(a,b)], reproduce the trends observed in steady-state measurements, including the hypsochromic shift for MoS_2_ and WS_2_ NSs/NPLs of 130 meV/40 nm and 20 meV/7 nm, respectively, and spectral broadening. To probe the dynamics, all samples were excited at their respective B-exciton energies (details in Figure S1) under a low excitation density of 4.7 μJ/cm^2^. The resulting carrier dynamics were extracted using global analysis of a sequential bi/triexponential model. This approach allows us to independently resolve decay times from overlapping signals while accounting for the instrument response function, ensuring accurate characterization of the sub-picosecond dynamics near the resolution limit^52^ (further described in the SI). As shown in Figure 2(c), the A exciton bleaches of MoS_2_ and WS_2_ NPLs exhibit a significantly faster decay compared to that of the larger NSs (see Table 1).

**Figure 2:**
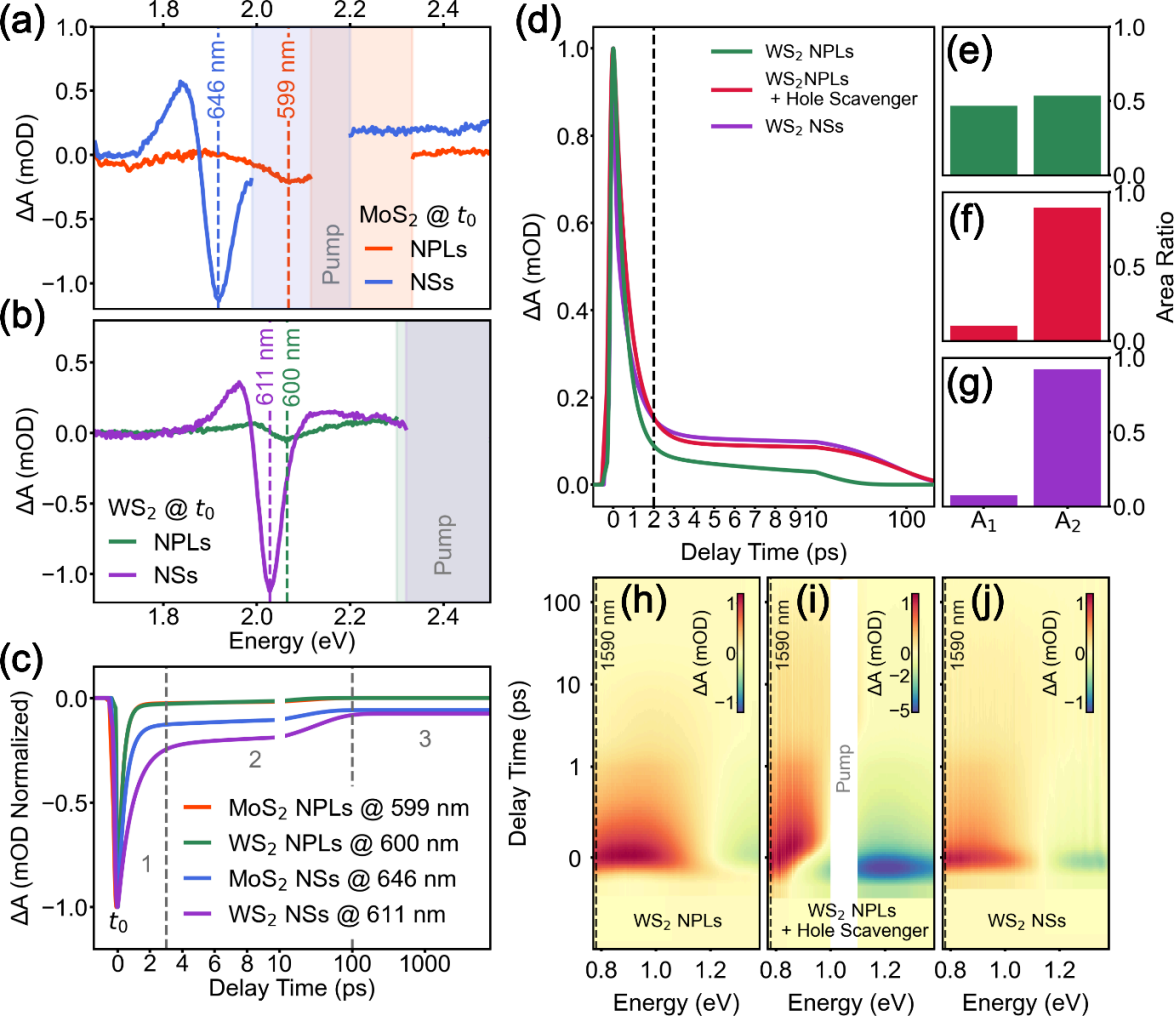
(a, b) Spectral line cuts of MoS_2_ (a) and WS_2_ (b) at *t*_0_ showcasing shift, broadening, and weakening of the A excitonic transition under lateral confinement. The spectral position of each A exciton is marked by a vertical line, while the areas obscured by the pump pulse are marked in the respective color of the corresponding graph. (c) Fitted decay dynamics of the A exciton of all samples. The time scale is linear between −0.5 and 10 ps and logarithmic from 10 to 7500 ps to allow both high resolution of the early response and maximum coverage. (d) Fitted decay traces of WS_2_ NPLs (green), WS_2_ NPLs with ascorbic acid added as hole scavenger (red), and WS_2_ NSs (purple). Integrated areas under each trace assigned to the first and second decay process (A_1_, A_2_) for (e) WS_2_ NPLs, (f) WS_2_ NPLs, and hole scavenger and (g) WS_2_ NSs, showing a reduced contribution of the initial trapping (first component, A_1_) when the hole scavenger is present. Associated hyperspectra showing WS_2_ NPLs (h), WS_2_ NPLs with hole scavenger (i), and WS_2_ NSs (j).

**Table 1:** Decay Times Fitted by a Sequential Bi/Tri-exponential Model for Initial Carrier Trapping *t*_1_, Bandgap Renormalization *t*_2_, and Slow Recombination from Trapped States *t*_3_ of MoS_2_ and WS_2_ NPLs/NSs

	*t*_1_	*t*_2_	*t*_3_
MoS_2_ NPLs	(270 ± 50) fs	(21 ± 7) ps	–
MoS_2_ NSs	(410 ± 100) fs	(40 ± 7) ps	→ ∞
WS_2_ NPLs	(291 ± 40) fs	(11 ± 1) ps	–
WS_2_ NSs	(741 ± 40) fs	(29 ± 2) ps	→ ∞

A rapid, sub-picosecond trapping process is observed in both MoS_2_ and WS_2_ nanostructures. However, as shown in Table 1, this effect is more pronounced in the NPLs, being faster and contributing more to the overall decay. Such a behavior points to a higher density of midgap trap states favoring nonradiative decay in NPLs.^7,28^ In monolayer colloidal TMD NSs, electron trapping mechanisms have been linked to sulfur vacancies,^30^ whereas hole trapping has been reported for multilayer NSs.^31^ These similar decay mechanisms cannot be directly translated to interpret the results obtained for the smaller MoS_2_ and WS_2_ NPLs.

The reduced lateral dimensions of NPLs lead to a significantly higher density of exposed edge sites compared with NSs, fundamentally altering exciton decay pathways. While both nanostructures show picosecond bathochromic absorption shifts consistent with bandgap renormalization^28,30,31,53^ (Figure S2), a distinct, long-lived process assigned to radiative recombination is observed exclusively in the larger NSs [Figure 2(c)], where μ-PL has been previously reported.^29,32,48^ In contrast, NPL dynamics are dominated by sub-picosecond trapping, which our measurements suggest is mediated by intrinsic midgap states. This accelerated nonradiative pathway effectively outcompetes radiative recombination, rationalizing the absence of detectable photoluminescence in the NPLs compared to their larger NS counterparts.

To isolate the excited carrier dynamics from ground-state absorption (Figure S3), we performed selective photoexcitation of the A and B transitions in WS_2_ and probed the near-infrared region. We observe a broad, positive excited-state absorption (ESA) signal [Figure 2(h–j)] assigned to intraband transitions,^54^ where the initial decay component reflects the trapping process observed in the visible range.^31^ Kinetic analysis along the 0.78 eV probe line reveals that trapping in NPLs is consistently faster (*Δt*_1_ ≈ 20 fs) upon A-exciton excitation, suggesting accelerated hole trapping when carriers are generated near the band edge (Figure S4). To confirm the hole-mediated nature of this process, we utilized ascorbic acid as a hole scavenger.^55^ The presence of the scavenger significantly extends the ESA decay lifetime [Figure 2(d)] and reduces the trapping contribution from 48% to ∼10% in NPLs [Figure 2(e–g)]. These findings conclusively identify holes as the primary drivers of the sub-picosecond trapping dynamics in WS_2_ NPLs.

To understand the midgap states inferred from our time-resolved spectroscopic measurements, we perform DFT calculations to investigate the electronic structure of a series of MoS_2_ and WS_2_ NPLs. Our model structures [Figure 3(a)] are monolayer-thick pseudohexagonal 2H-polymorphic NPLs with varying lateral dimensions of 0.9, 1.5, and 2.1 nm. These model structures are qualitatively representative of the experimental NPLs reported in Figure 1(a). Their size falls in the experimental range, which is reasonable for rationalizing the measurements. The optimized geometries are defect-free and fully passivated, featuring zigzag (ZZ, edge S-atoms bonded to two Mo (W) atoms) and armchair (AC, edge S-atoms bonded to a single Mo (W) atom) edges. Hydrogen passivation effectively removes spurious midgap states from undercoordinated S-atoms, while preserving the dominant electronic characteristics dictated by the edge species.^56^ It is also worth noting that H-terminated colloidal TMD nanostructures have been reported in the experimental literature.^57–61^

**Figure 3:**
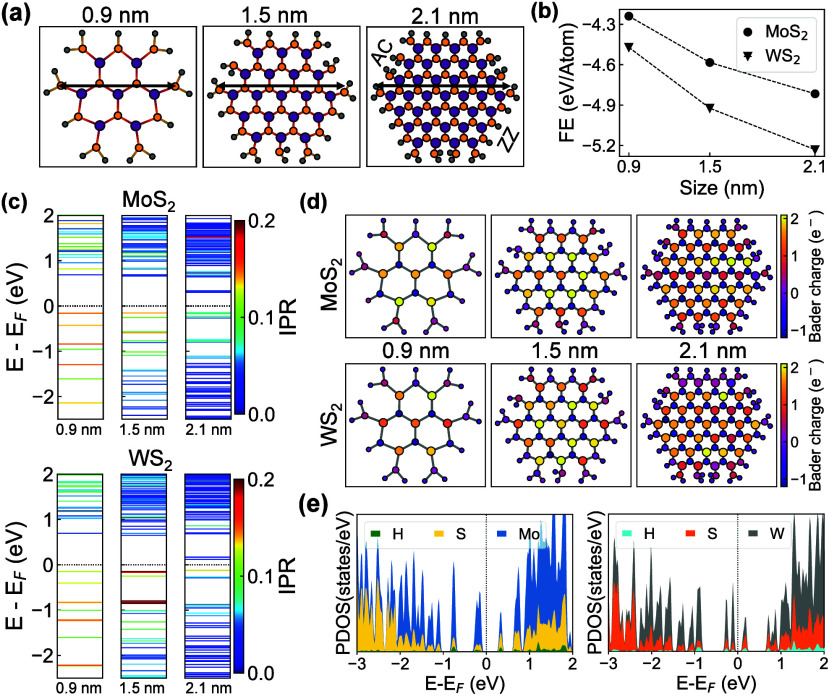
(a) Optimized structures of hydrogen-passivated MoS_2_ nanoplatelets (NPLs) of different sizes with zigzag (ZZ) and armchair (AC) S-edges. Mo atoms are colored purple, S yellow, and H gray. Black arrows indicate the size. (b) Formation energies of MoS_2_ and WS_2_ NPLs as a function of size. (c) Inverse participation ratio for states around the Fermi energy (*E*_F_) for MoS_2_ and WS_2_ NPLs. (d) Net Bader charges for calculated MoS_2_ (top) and WS_2_ (bottom) NPL sizes. (e) Projected density of states (PDOS) near the Fermi level for 2.1 nm MoS_2_ (left) and WS_2_ (right) NPLs.

The formation energy [Figure 3(b)] decreases as both the MoS_2_ and WS_2_ NPL size grows. This trend is consistent with the size distribution observed in our measurements [Figure 1(c)] and reflects the enhanced thermodynamic stability of larger NPLs. At comparable sizes, WS_2_ NPLs are consistently more stable than MoS_2_, due to the stronger bonds and higher ionic character of W–S relative to Mo–S bond.^62^

To verify whether trap states are present in the NPLs, we investigate the electronic structure [Figure 3(c)] near the Fermi level (*E*_F_). Both MoS_2_ and WS_2_ NPLs show semiconducting behavior with a bandgap decreasing as the NPL size increases, following the expected trend of quantum confinement. The single-particle-state analysis based on the inverse participation ratio^46,63,64^ [IPR, Figure 3(c)], a standard metric to assess wave function localization,^65,66^ shows that several hole (occupied) states near *E*_F_ in both WS_2_ and MoS_2_ have high IPR values across all considered sizes, indicating a strong localization. In the smallest structures (0.9 nm) of both materials, most hole states are relatively localized compared with the largest NPL (2.1 nm). Conversely, the deeper occupied states become increasingly delocalized, approaching a bulk-like character. This trend is a size effect resulting from the decreased relative number of edge atoms in larger flakes. In contrast, the electron (unoccupied) states remain largely delocalized, with only occasional localized states in the largest MoS_2_ and WS_2_ NPLs. Notably, WS_2_ NPLs show more localized states near *E*_F_ than MoS_2_, which correlates with the differences in the temporal measurements of the energy shift. The strongly localized states in WS_2_ have energies that are only weakly dependent on their lateral size, resulting in a much smaller shift of 20 meV.

To analyze the origin of electronic state localization, we perform Bader charge analysis of local charge fluctuations in the considered structures [Figure 3(d)]. Each NPL has a uniform core and a chemically distinct edge (Mo–S–H or W–S–H), with charge deviations increasing in larger flakes (Figure 3(d)]. In the central region, electron transfer from Mo (or W) to S occurs, consistent with their different electronegativity. At the edges, disrupted bonding reduces the ability of atoms to fully share or accept electrons and breaks the local lattice periodicity, giving rise to spatially localized electronic states [Figure 3(c)] near *E*_F_. Despite S-termination and H-passivation, edge metal atoms dominate the charge distribution, indicating their major role in the localized states. Different charge fluctuation patterns emerge in MoS_2_ and WS_2_ NPLs of equal size. The central metal atoms in WS_2_ display a more uniform charge distribution, in contrast to the uneven charge distribution in MoS_2_, which also leads to charge differences at the edges. The projected density of states (PDOS) of MoS_2_ and WS_2_ NPLs provides additional information about the orbital contributions to the energy levels. As shown in Figure 3(e) for the systems with 2.1 nm size, the hole (occupied) states up to about 2 eV below the Fermi level are dominated by metal *d*-orbitals (Figure S5), while S *p*-orbitals contribute deeper in energy, although signs of hybridization are present everywhere. In the electron (unoccupied) region, the trend does not change significantly, with the metal *d*-orbitals remaining predominant in the considered energy range. Here, sulfur states contribute more than those in the hole region.

We complete our analysis by inspecting the contributions from the interior (core) and exterior (edge) regions of the considered NPLs [Figure 4]. Specifically, we identify the origin of a specific state if more than 50% of its contributions comes from either portion of the nanostructure. Across all structures and in both compositions, both hole and electron states near *E*_F_ are predominantly edge-localized across all sizes of MoS_2_ and WS_2_ [Figure 4(a,b)]. In the smallest flake (0.9 nm), where nearly all of the atoms lie at the periphery, all states mainly originate from the edges. As the flake size increases, core-localized states gradually emerge; however, even in the largest flake (2.1 nm), states within ∼1 eV of the Fermi level remain mostly edge-localized.

**Figure 4:**
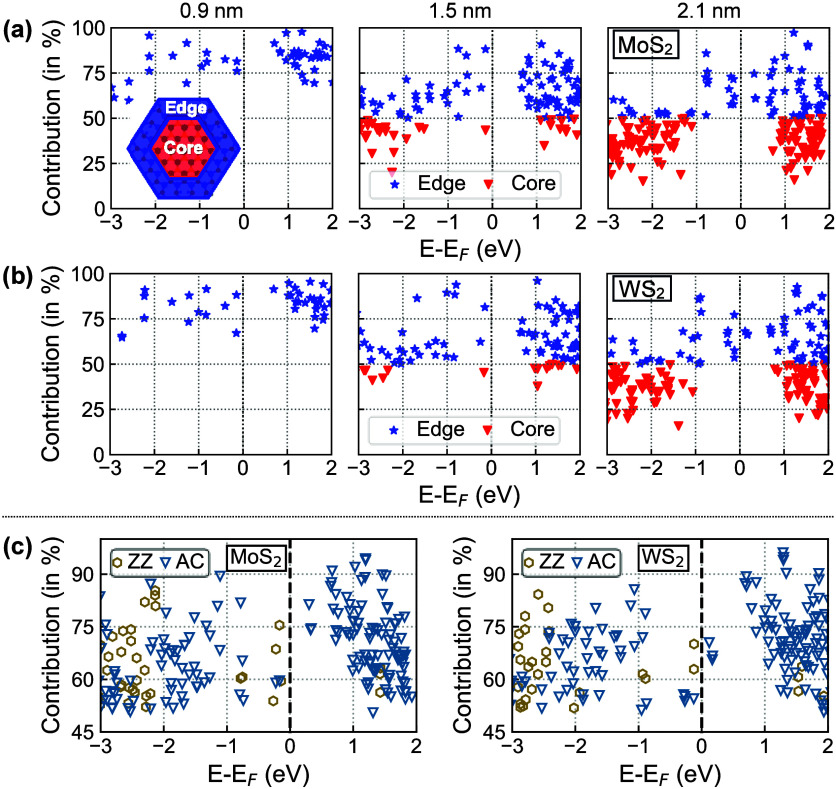
Calculated core–edge contributions to energy levels near the Fermi energy in (a) MoS_2_ and (b) WS_2_ NPLs. Blue stars (red triangles) mark states with >50% edge (core) contribution for the core–edge partitioning. (c) Relative contributions of zigzag (ZZ) and armchair (AC) edges to electronic states near the Fermi energy (*E*_F_) in 2.1 nm MoS_2_ (left) and (b) WS_2_ (right) NPLs.

For the largest, 2.1 nm NPLs, we additionally investigate the contributions of states belonging to each edge type (Figure 4(c)]. Near *E*_F_ in the occupied region, states on ZZ edges contribute slightly more than those on AC edges, while deeper hole states show increased contributions from AC edges. Electron states up to ∼1 eV are mostly dominated by AC-edge contributions in both MoS_2_ and WS_2_ NPLs. This edge-dependent behavior, characterized by localized states and charge imbalances, establishes the edges as chemically active sites. These findings thus explain why MoS_2_ and WS_2_ nanostructures are highly effective in photocatalysis.^67–74^

To gain qualitative insight into the optical activity of the investigated NPLs, we calculate the normalized oscillator strengths within the independent-particle approximation (IPA); see Figure 5. Consistent with the prevalence of edge states in the gap region of both WS_2_ and MoS_2_ NPLs, the lowest-energy transitions primarily involve edge-localized states with no core-originated transitions appearing below 4 eV in the spectrum of the smallest flake (0.9 nm). As the flake size increases to 2.1 nm, core-localized transitions progressively emerge but they remain systematically higher in energy compared to the edge-localized excitations, in agreement with the localization of the electronic states near the Fermi energy. While in both MoS_2_ and WS_2_ 1.5 nm NPLs manifolds of weak core-originated transitions appear in Figure 5(a,b), in the nanostructures with 2.1 nm radius, the density of these excited states and their relative strength increase dramatically, contributing substantially to the optical activities of both NPLs above 2 eV.

**Figure 5:**
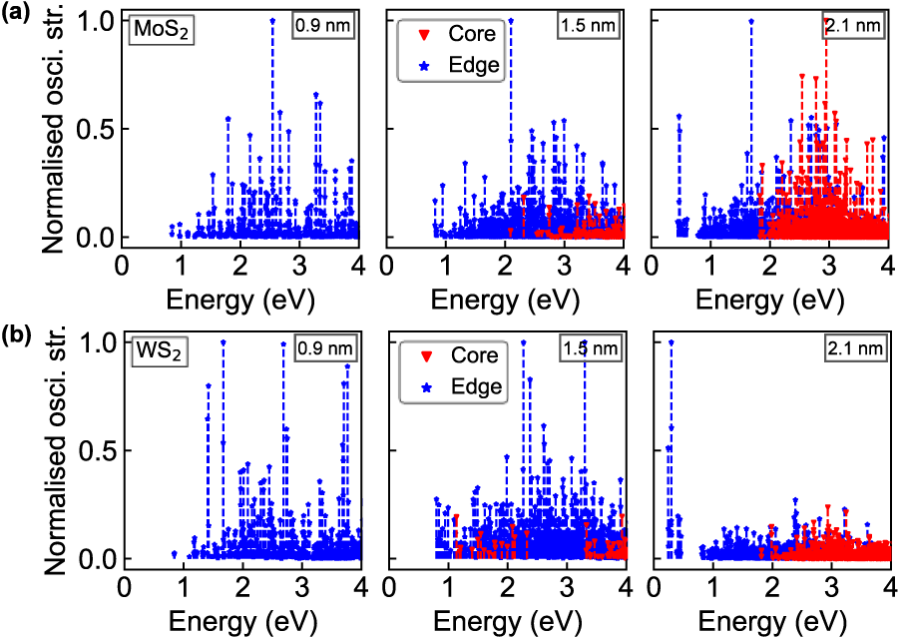
Calculated oscillator strengths for transitions below 4 eV in (a) MoS_2_ and (b) WS_2_ NPLs, colored based on state character: red (core-originated, both states <50% edge character), blue (edge-originated, at least one state has >50% edge character).

The observed size dependence of these transitions demands a thorough comparison with the excitonic properties of extended TMDs. In these materials, the exciton Bohr radius typically ranges from 1 to 2 nm, which is comparable to the lateral dimensions of our NPLs. While conventional effective-mass models might predict similar lateral confinement effects for both materials, our results highlight a qualitative divergence. In MoS_2_, the transitions exhibit a “particle-in-a-box” behavior with a strong size sensitivity (130 meV blue shift), indicating that the excitons are indeed laterally confined within the core. In contrast, the optical response in WS_2_ is dominated by edge-localized states, which are intrinsically less sensitive to quantum dot size. Hence, their energies are effectively pinned by the edge potential, showing only a 20 meV shift and effectively suppressing the expected size dependence of the primary optical transition.

These findings suggest that core-originated transitions will dominate over edge transitions in larger NSs, leading to longer-lived exciton decay,^28^ consistent with the measured decay times summarized in Table 1. Direct comparison of MoS_2_ and WS_2_ NPLs reveals markedly enhanced edge-state emission in WS_2_. In 2.1 nm WS_2_, the edge-originated excited states at the spectral onset (0.2–0.5 eV) are ∼10 orders of magnitude brighter than the higher-energy core transitions, while in MoS_2_, the relative intensity of the two manifolds is comparable. This striking contrast signifies stronger optical coupling and radiative recombination in WS_2_, consistent with our measurements. Physically, this is due to the larger spatial confinement and higher transition dipole moments of the *W*-dominated edge states. The larger spin–orbit coupling and more localized 5*d*-orbitals of W, compared to Mo, enhance the separation between the edge-trap manifold and the core continuum. Consequently, WS_2_ nanostructures exhibit higher intrinsic radiative rates at the edges (Figure 2) where excitons possess relatively high oscillator strengths (Figure 5b). In contrast, in MoS_2_, the comparable core- and edge-state intensities promote state hybridization and nonradiative quenching, which also agrees with the findings.

Furthermore, our work contextualizes different carrier trapping as compared to larger TMD nanosheets.^30^ In MoS_2_ and WS_2_ NPLs, we identify that lateral edges control the optical response which host optically bright hole traps (majorly) from highly localized Mo/W *d*-orbitals. While the high oscillator strength of these states suggests the potential for subgap emission, consistent with subgap features often attributed to defect-bound excitons or trions in larger TMD nanosheets,^32,75^ we do not observe PL in the smallest NPLs. This can be understood as a kinetic competition where, despite the bright nature of the edge transitions, the high density of surface states and vibrational modes in these high-surface-area structures provides dominant nonradiative decay channels. Consequently, the carrier population is quenched on a sub-picosecond time scale, effectively overshadowing the intrinsic optical activity of these edge states.

Finally, these DFT results provide a physical basis to rationalize the experimental decay components in Table 1. The longest component, uniquely observed in the larger NSs, can be assigned to radiative recombination from core-originated states. In the smaller NPLs, where the optical response is dominated by edge-localized states, bright edge traps lead to ultrafast quenching of the core-exciton population. Hence, the nanosecond radiative component vanishes in the NPL limit, where the maximized edge-to-core ratio ensures that the nonradiative trapping rate effectively outpaces radiative recombination. This correlation is further evidenced by the absence of detectable PL in NPLs, in contrast to the sizable emission observed in the NSs.

In summary, this study offers a comprehensive understanding of the exciton dynamics in colloidal WS_2_ and MoS_2_ NPLs, revealing the atomistic origin of their quenched PL. We demonstrate edge-located hole traps to explain the ultrafast sub-picosecond exciton decay in laterally confined TMD NPLs, even without the introduction of sulfur vacancies. Our combined experimental and theoretical approach identifies these traps as optically bright. With an increasing NPL size, the core steadily dominates, reducing the contribution of edge states and resulting in longer-lived exciton decay, consistent with our experimental observations on NSs. Furthermore, a high density of localized states at the edges enables efficient charge transfer. This explains the exceptional photocatalytic activity of the TMD nanostructures.

Our work unravels not only the fundamental physics behind exciton quenching in colloidal TMDs but also opens a pathway for future design strategies, like postsynthesis chemical treatment or functionalization, to optimize the optical behavior of these systems. With the gained understanding of the importance of metal *d*-states and their influence of NPL size and edge shape, we can now explore new avenues to enhance PL quantum efficiency and catalytic performance. We anticipate that the trap states predicted here should also be manifested in other TMD nanostructures. Future work based on the solution of the Bethe–Salpeter equation, including all electron–electron and electron–hole correlation mechanisms properly describing excitons, will provide quantitative characterization of the excited states and their dynamics. Likewise, the inclusion of explicit ligands will improve our understanding of the impact of functionalization on the PL efficiency.

## Supplementary Material



## Data Availability

Input and output files of the *ab initio* calculations performed in this work are available free of charge in Zenodo DOI: https://doi.org/10.5281/zenodo.17661456 [record: 17661456]. Experimental data are available from the corresponding author J.L. upon reasonable request.

## Notes

Experimental and computational method details; structural properties, element and orbital resolved PDOS for all structures from the main text (PDF)

## References

[1] Mahler B., Hoepfner V., Liao K., Ozin G. A. (2014). Colloidal Synthesis
of 1T-WS_2_ and 2H-WS_2_ Nanosheets: Applications
for Photocatalytic Hydrogen Evolution. J. Am.
Chem. Soc..

[2] Sun Y., Fujisawa K., Lin Z., Lei Y., Mondschein J. S., Terrones M., Schaak R. E. (2017). Low-Temperature
Solution Synthesis
of Transition Metal Dichalcogenide Alloys with Tunable Optical Properties. J. Am. Chem. Soc..

[3] Sun Y., Terrones M., Schaak R. E. (2021). Colloidal Nanostructures of Transition-Metal
Dichalcogenides. Acc. Chem. Res..

[4] Chen C., Qiao H., Lin S., Man Luk C., Liu Y., Xu Z., Song J., Xue Y., Li D., Yuan J., Yu W., Pan C., Ping Lau S., Bao Q. (2015). Highly Responsive MoS_2_ Photodetectors Enhanced by Graphene Quantum Dots. Sci. Rep..

[5] Zechel F., Hutár P., Vretenár V., Végsö K., Šiffalovič P., Sýkora M. (2023). Green Colloidal
Synthesis of MoS_2_ Nanoflakes. Inorg.
Chem..

[6] Kapuria N., Patil N. N., Sankaran A., Laffir F., Geaney H., Magner E., Scanlon M., Ryan K. M., Singh S. (2023). Engineering
Polymorphs in Colloidal Metal Dichalcogenides: Precursor-Mediated
Phase Control, Molecular Insights into Crystallisation Kinetics and
Promising Electrochemical Activity. J. Mater.
Chem. A.

[7] Niebur A., Söll A., Haizmann P., Strolka O., Rudolph D., Tran K., Renz F., Frauendorf A. P., Hübner J., Peisert H., Scheele M., Lauth J. (2023). Untangling
the Intertwined: Metallic to Semiconducting Phase Transition of Colloidal
MoS_2_ Nanoplatelets and Nanosheets. Nanoscale.

[8] Fröhlich M., Kögel M., Hiller J., Kahlmeyer L., Meixner A. J., Scheele M., Meyer J. C., Lauth J. (2024). Colloidal
2D Mo_1–*x*
_W_
*x*
_S_2_ Nanosheets: An Atomic- to Ensemble-level Spectroscopic
Study. Phys. Chem. Chem. Phys..

[9] Joseph S., Mohan J., Lakshmy S., Thomas S., Chakraborty B., Thomas S., Kalarikkal N. (2023). A Review of
the Synthesis, Properties,
and Applications of 2D Transition Metal Dichalcogenide and their Heterostructures. Mater. Chem. Phys..

[10] Gopalakrishnan D., Damien D., Li B., Gullappalli H., Pillai V. K., Ajayan P. M., Shaijumon M. M. (2015). Electrochemical
Synthesis of Luminescent MoS_2_ Quantum Dots. Chem. Commun..

[11] Wang X., Sun G., Li N., Chen P. (2016). Quantum Dots Derived from Two-dimensional
Materials and their Applications for Catalysis and Energy. Chem. Soc. Rev..

[12] Nguyen V., Dong Q., Yan L., Zhao N., Le P. H. (2019). Facile
Synthesis of Photoluminescent MoS_2_ and WS_2_ Quantum
Dots with Strong Surface-state Emission. J.
Lumin..

[13] Golovynskyi S., Bosi M., Seravalli L., Li B. (2021). MoS_2_ Two-dimensional
Quantum Dots with Weak Lateral Quantum Confinement: Intense Exciton
and Trion Photoluminescence. Surf. Interfaces.

[14] Doolen R., Laitinen R., Parsapour F., Kelley D. F. (1998). Trap State Dynamics
in MoS_2_ Nanoclusters. J. Phys. Chem.
B.

[15] Gan Z. X., Liu L. Z., Wu H. Y., Hao Y. L., Shan Y., Wu X. L., Chu P. K. (2015). Quantum
Confinement Effects across
Two-dimensional Planes in MoS_2_ Quantum Dots. Appl. Phys. Lett..

[16] Li B., Jiang L., Li X., Ran P., Zuo P., Wang A., Qu L., Zhao Y., Cheng Z., Lu Y. (2017). Preparation of Monolayer MoS_2_ Quantum Dots using Temporally
Shaped Femtosecond Laser Ablation of Bulk MoS_2_ Targets
in Water. Sci. Rep..

[17] Pallikkarathodi
Mani N., Cyriac J. (2020). Hydrothermal Synthesis of WS_2_ Quantum Dots
and their Application as a Fluorescence Sensor for the Selective Detection
of 2,4,6-trinitrophenol. New J. Chem..

[18] Yin W., Bai X., Chen P., Zhang X., Su L., Ji C., Gao H., Song H., Yu W. W. (2018). Rational Control of Size and Photoluminescence
of WS_2_ Quantum Dots for White Light-Emitting Diodes. ACS Appl. Mater. Interfaces.

[19] Chiu C.-H., Chen Y.-T., Shen J.-L. (2023). Quantum
Dots Derived from Two-dimensional
Transition Metal Dichalcogenide: Synthesis, Optical Properties and
Optoelectronic Applications. Nanotechnology.

[20] Bertram N., Cordes J., Kim Y., Ganteför G., Gemming S., Seifert G. (2006). Nanoplatelets Made
from MoS_2_ and WS_2_. Chem.
Phys. Lett..

[21] Mak K. F., Lee C., Hone J., Shan J., Heinz T. F. (2010). Atomically Thin
MoS_2_: A New Direct-gap Semiconductor. Phys. Rev. Lett..

[22] Wang Q. H., Kalantar-Zadeh K., Kis A., Coleman J. N., Strano M. S. (2012). Electronics
and Optoelectronics of Two-dimensional Transition Metal Dichalcogenides. Nat. Nanotechnol..

[23] Chernikov A., Berkelbach T. C., Hill H. M., Rigosi A., Li Y., Aslan B., Reichman D. R., Hybertsen M. S., Heinz T. F. (2014). Exciton Binding Energy and Nonhydrogenic Rydberg Series
in Monolayer WS_2_. Phys. Rev. Lett..

[24] Brokmann X., Coolen L., Dahan M., Hermier J. P. (2004). Measurement of the
Radiative and Nonradiative Decay Rates of Single CdSe Nanocrystals
through a Controlled Modification of their Spontaneous Emission. Phys. Rev. Lett..

[25] Nirmal M., Dabbousi B. O., Bawendi M. G., Macklin J. J., Trautman J. K., Harris T. D., Brus L. E. (1996). Fluorescence Intermittency in Single
Cadmium Selenide Nanocrystals. Nature.

[26] Durisic N., Wiseman P. W., Grütter P., Heyes C. D. (2009). A Common Mechanism
Underlies the Dark Fraction Formation and Fluorescence Blinking of
Quantum Dots. ACS Nano.

[27] Banin U., Bruchez M., Alivisatos A. P., Ha T., Weiss S., Chemla D. S. (1999). Evidence for a Thermal Contribution
to Emission Intermittency
in Single CdSe/CdS Core/shell nanocrystals. J. Chem. Phys..

[28] Frauendorf A. P., Niebur A., Rudolph D., Oestreich M., Lauth J., Hübner J. (2024). Ultrafast Recombination Dynamics
under Lateral Confinement and Cryogenic Temperatures in Colloidal
MoS_2_. J. Phys. Chem. C.

[29] Pippia G., Rousaki A., Barbone M., Billet J., Brescia R., Polovitsyn A., Martín-García B., Prato M., De Boni F., Petrić M. M., Ben Mhenni A., Van Driessche I., Vandenabeele P., Müller K., Moreels I. (2022). Colloidal Continuous
Injection Synthesis
of Fluorescent MoX_2_ (X = S, Se) Nanosheets as a First Step
Toward Photonic Applications. ACS Appl. Nano
Mater..

[30] Mutyala C. S., Pippia G., Tanghe I., Martín-García B., Rousaki A., Vandenabeele P., Schiettecatte P., Moreels I., Geiregat P. (2023). Charge Carrier Dynamics in Colloidally
Synthesized Monolayer MoX_2_ Nanosheets. J. Phys. Chem. Lett..

[31] Schiettecatte P., Geiregat P., Hens Z. (2019). Ultrafast Carrier Dynamics
in Few-Layer
Colloidal Molybdenum Disulfide Probed by Broadband Transient Absorption
Spectroscopy. J. Phys. Chem. C.

[32] Zhao Y., Fröhlich M., Kögel M., Strolka O., Niebur A., Parker T., Schneider F., Meixner A. J., Meyer J. C., Zhang D., Lauth J. (2025). Second-harmonic Generation and Photoluminescence
Properties of Colloidal WS_2_ Monolayers Deposited from Solution. Nanoscale Horiz..

[33] Cunningham P. D., McCreary K. M., Hanbicki A. T., Currie M., Jonker B. T., Hayden L. M. (2016). Charge Trapping and Exciton Dynamics in Large-Area
CVD Grown MoS_2_. J. Phys. Chem. C.

[34] Li H., Wu J., Yin Z., Zhang H. (2014). Preparation and Applications of Mechanically
Exfoliated Single-layer and Multilayer MoS_2_ and WSe_2_ Nanosheets. Acc. Chem. Res..

[35] Eda G., Yamaguchi H., Voiry D., Fujita T., Chen M., Chhowalla M. (2011). Photoluminescence
from Chemically Exfoliated MoS_2_. Nano Lett..

[36] Nicolosi V., Chhowalla M., Kanatzidis M. G., Strano M. S., Coleman J. N. (2013). Liquid
Exfoliation of Layered Materials. Science.

[37] Lee Y.-H., Zhang X.-Q., Zhang W., Chang M.-T., Lin C.-T., Chang K.-D., Yu Y.-C., Wang J. T.-W., Chang C.-S., Li L.-J., Lin T.-W. (2012). Synthesis
of Large-Area MoS_2_ Atomic Layers with Chemical Vapor Deposition. Adv. Mater..

[38] Zhao W., Ghorannevis Z., Chu L., Toh M., Kloc C., Tan P.-H., Eda G. (2013). Evolution of Electronic Structure
in Atomically Thin Sheets of WS_2_ and WSe_2_. ACS Nano.

[39] Mukherjee S., NM A. K., Mondal A., Mahalingam V., Kamaraju N. (2023). Trapping and Exciton-Exciton Annihilation Assisted
Ultrafast Carrier Dynamics in Nanosheets of 2H-MoSe_2_ and
Cr Doped 1T/2H-MoSe_2_. J. Chem. Phys..

[40] Wang H., Strait J. H., Zhang C., Chan W., Manolatou C., Tiwari S., Rana F. (2015). Fast Exciton
Annihilation by Capture
of Electrons or Holes by Defects via Auger Scattering in Monolayer
Metal Dichalcogenides. Phys. Rev. B.

[41] Zhang S., Hill H. M., Moudgil K., Richter C. A., Hight
Walker A. R., Barlow S., Marder S. R., Hacker C. A., Pookpanratana S. J. (2018). Controllable, Wide-Ranging n-Doping and p-Doping of
Monolayer Group 6 Transition-Metal Disulfides and Diselenides. Adv. Mater..

[42] Houtepen A. J., Hens Z., Owen J. S., Infante I. (2017). On the Origin of Surface
Traps in Colloidal II-VI Semiconductor Nanocrystals. Chem. Mater..

[43] Giansante C., Infante I. (2017). Surface Traps in Colloidal Quantum
Dots: A Combined
Experimental and Theoretical Perspective. J.
Phys. Chem. Lett..

[44] Kirkwood N., Monchen J. O. V., Crisp R. W., Grimaldi G., Bergstein H. A. C., du Fossé I., van der Stam W., Infante I., Houtepen A. J. (2018). Finding and Fixing Traps in II-VI
and III-V Colloidal Quantum Dots: The Importance of Z-Type Ligand
Passivation. J. Am. Chem. Soc..

[45] Alexander E., Kick M., McIsaac A. R., Van Voorhis T. (2024). Understanding
Trap States in InP and GaP Quantum Dots through Density Functional
Theory. Nano Lett..

[46] Kumar S., Cocchi C., Steenbock T. (2025). Surface Defects
and Symmetry Breaking
Impact on the Photoluminescence of InP Quantum Dots. Nano Lett..

[47] Pippia G., Van Hamme D., Martín-García B., Prato M., Moreels I. (2022). A Colloidal Route to Semiconducting
Tungsten Disulfide Nanosheets with Monolayer Thickness. Nanoscale.

[48] Frauendorf A. P., Niebur A., Harms L., Shree S., Urbaszek B., Oestreich M., Hübner J., Lauth J. (2021). Room Temperature Micro-Photoluminescence
Studies of Colloidal WS_2_ Nanosheets. J. Phys. Chem. C.

[49] Fouladi-Oskouei J., Shojaei S., Liu Z. (2018). Robust Tunable Excitonic
Features
in Monolayer Transition Metal Dichalcogenide Quantum Dots. J. Phys.: Condens. Matter.

[50] Ayari S., Quick M. T., Owschimikow N., Christodoulou S., Bertrand G. H. V., Artemyev M., Moreels I., Woggon U., Jaziri S., Achtstein A. W. (2020). Tuning
Trion Binding Energy and Oscillator
Strength in a Laterally Finite 2D System: CdSe Nanoplatelets as a
Model System for Trion Properties. Nanoscale.

[51] Rodà C., Geiregat P., Di Giacomo A., Moreels I., Hens Z. (2022). Area-Independence
of the Biexciton Oscillator Strength in CdSe Colloidal Nanoplatelets. Nano Lett..

[52] Frech P., Scheele M. (2026). TAPAS: Transient Absorption Processing
and Analysis
Software. ChemPhotoChem..

[53] Pogna E. A. A., Marsili M., De Fazio D., Dal Conte S., Manzoni C., Sangalli D., Yoon D., Lombardo A., Ferrari A. C., Marini A., Cerullo G., Prezzi D. (2016). Photo-Induced
Bandgap Renormalization Governs the Ultrafast Response of Single-Layer
MoS_2_. ACS Nano.

[54] Wang H., Zhang C., Rana F. (2015). Ultrafast
Dynamics of Defect-Assisted
Electron-Hole Recombination in Monolayer MoS_2_. Nano Lett..

[55] Dunklin J. R., Zhang H., Yang Y., Liu J., van de Lagemaat J. (2018). Dynamics of
Photocatalytic Hydrogen Production in Aqueous Dispersions of Monolayer-Rich
Tungsten Disulfide. ACS Energy Letters.

[56] Krumland J., Valencia A. M., Cocchi C. (2021). Exploring
Organic Semiconductors
in Solution: the Effects of Solvation, Alkylization, and Doping. Phys. Chem. Chem. Phys..

[57] Sharifvaghefi S., Yang B., Zheng Y. (2018). New Insights on the Role of H_2_S and Sulfur Vacancies on Dibenzothiophene Hydrodesulfurization
over MoS_2_ Edges. Appl. Catal. A:
Gen..

[58] Lauritsen J. V., Nyberg M., Vang R. T., Bollinger M. V., Clausen B. S., Topsøe H., Jacobsen K. W., Lægsgaard E., Nørskov J. K., Besenbacher F. (2003). Chemistry of One-dimensional Metallic
Edge States in MoS_2_ Nanoclusters. Nanotechnology.

[59] Topsoe N., Topsoe H. (1993). FTIR Studies of Mo/Al_2_O_3_-Based
Catalysts: II. Evidence for the Presence of SH Groups and Their Role
in Acidity and Activity. J. Catal..

[60] Ma K. Y., Yoon S. I., Jang A.-R., Jeong H. Y., Kim Y.-J., Nayak P. K., Shin H. S. (2017). Hydrogenation
of Monolayer Molybdenum
Diselenide via Hydrogen Plasma Treatment. J.
Mater. Chem. C.

[61] Din N. U., Turkowski V., Rahman T. S. (2021). Excited States in Hydrogenated Single-layer
MoS_2_. J. Phys.: Condens. Matter.

[62] Pandey D., Chakrabarti A. (2019). Prediction
of Two-dimensional Monochalcogenides: MoS
and WS. Phys. Lett. A.

[63] Calixto M., Romera E. (2015). Inverse Participation
Ratio and Localization in Topological
Insulator. Theory Exp.

[64] Steenbock T., Drescher E., Dittmann T., Bester G. (2024). How Surface Defects
Shape the Excitons and Photoluminescence of Ultrasmall CdSe Quantum
Dots. Chem. Mater..

[65] Niebur A., Lorenz T., Schreiber M., Seifert G., Gemming S., Joswig J.-O. (2021). Localization of
Edge States at Triangular Defects in
Periodic MoS_2_ Monolayers. Phys. Rev.
Mater..

[66] Niebur A., Lorenz T., Joswig J.-O., Seifert G., Gemming S., Schreiber M. (2021). Closed-Loop
Defect States in 2D Materials with Honeycomb
Lattice Structure: Molybdenum Disulfide. Phys.
Status Solidi B.

[67] Yuan Y.-J., Yu Z.-T., Liu X.-J., Cai J.-G., Guan Z.-J., Zou Z.-G. (2014). Hydrogen Photogeneration Promoted by Efficient Electron
Transfer from Iridium Sensitizers to Colloidal MoS_2_ Catalysts. Sci. Rep..

[68] Kayal A., De M. (2024). Organo-Soluble Colloidal
MoS2 Quantum Dots (QDs) as an Efficient
Photocatalyst for *α*-Amino Phosphonate Synthesis. ChemCatChem..

[69] Rahman A., Jennings J. R., Tan A. L., Khan M. M. (2022). Molybdenum Disulfide-Based
Nanomaterials for Visible-Light-Induced Photocatalysis. ACS Omega.

[70] Li Z., Meng X., Zhang Z. (2018). Recent Development on MoS_2_-based Photocatalysis: A Review. J. Photochem.
Photobiol., C.

[71] Yuan Y.-J., Lu H.-W., Yu Z.-T., Zou Z.-G. (2015). Noble-Metal-Free
Molybdenum Disulfide Cocatalyst for Photocatalytic Hydrogen Production. ChemSusChem.

[72] Jin X., Fan X., Tian J., Cheng R., Li M., Zhang L. (2016). MoS_2_ Quantum dot Decorated g-C_3_N_4_ Composite Photocatalyst
with Enhanced Hydrogen Evolution Performance. RSC Adv..

[73] Xiao P., Lou J., Zhang H., Song W., Wu X.-L., Lin H., Chen J., Liu S., Wang X. (2018). Enhanced Visible-light-driven
Photocatalysis from WS_2_ Quantum Dots Coupled to BiOCl Nanosheets:
Synergistic Effect and Mechanism Insight. Catal.
Sci. Technol..

[74] Zou Y., Shi J.-W., Ma D., Fan Z., Cheng L., Sun D., Wang Z., Niu C. (2018). WS_2_/Graphitic Carbon Nitride
Heterojunction Nanosheets Decorated with CdS Quantum Dots for Photocatalytic
Hydrogen Production. ChemSusChem.

[75] Chow P. K., Jacobs-Gedrim R. B., Gao J., Lu T.-M., Yu B., Terrones H., Koratkar N. (2015). Defect-Induced
Photoluminescence
in Monolayer Semiconducting Transition Metal Dichalcogenides. ACS Nano.

