# Supplementary material for: Atomistic Origin of Photoluminescence Quenching in Colloidal MoS2 and WS2 Nanoplatelets
Source: Nano Lett. 2026 Feb 28;26(9):3064–72. doi: 10.1021/acs.nanolett.5c05893 (PMC12983361; doi:10.1021/acs.nanolett.5c05893)
Supplement: Supplementary file 1 [file nl5c05893_si_001.pdf]

SUPPORTING INFORMATION: Atomistic Origin of Photoluminescence Quenching in Colloidal MoS_2 and WS_2 Nanoplatelets

Surender Kumar,[*,†,#] Markus Fröhlich,[‡,#] Stefan Velja,[†] Marco Kögel,[¶,§] Onno Strolka,[‡,∥] André Niebur,[∥] Samuell Ginzburg,[⊥] Muhammad Sufyan Ramzan,[†] Jannik C. Meyer,[§,¶] Jannika Lauth,[*,‡,∥] and Caterina Cocchi[*,†]

†*Institut für Festkörpertheorie und -Optik, Friedrich-Schiller-Universität Jena, 07743 Jena, Germany*

‡*Institute of Physical and Theoretical Chemistry, Eberhard Karls University of Tübingen, 72076 Tübingen, Germany*

¶*NMI Natural and Medical Sciences Institute at the University of Tübingen, 72770 Reutlingen, Germany*

§*University of Tübingen, Institute of Applied Physics, 72076 Tübingen, Germany*

∥*Leibniz University of Hannover, Cluster of Excellence PhoenixD (Photonics, Optics and Engineering - Innovation Across Dimensions), 30167 Hannover, Germany*

⊥*Yusuf Hamied Department of Chemistry, University of Cambridge, Lensfield Road, CB2 1EW Cambridge, UK*

#*These authors contributed equally to this work.*

E-mail: surendermohinder@gmail.com; jannika.lauth@uni-tuebingen.de; caterina.cocchi@uni-jena.de

Experimental Methods

Transmission Electron Microscopy (TEM)

HR-TEM images were collected using a JEOL ARM 200F equipped with a CETCOR aberration corrector operating at 80 kV. Samples were prepared by drop-casting the WS_2 NPL or MoS_2 NS solution (in hexane) onto graphene-coated MEMS (Graphenea easy-transfer) heating chips purchased from Hennyz Solutions. The dry sample was cleaned subsequently by flushing of the TEM-grid with hexane and ethanol. To remove mobile carbon contamination, the sample was heated to 300 °C under high vacuum conditions of the electron microscope.

UV-Vis Absorption Spectroscopy

UV-Vis absorption spectra were acquired on an Agilent Cary 5000 equipped with a diffuse reflectance accessory in transmission mode using a quartz cuvette (quartz glass high performance QS200–2500 nm by Hellma) with a path length of 1 cm. All samples were dispersed in ortho-dicholorbenzene (o-DCB) for maximum colloidal stability.

Transient absorption spectroscopy (TAS)

Transient absorption spectra were obtained using a spectral broadband femtosecond transient absorption spectrometer (Ultrafast Systems, HELIOS Fire). A Ti/Sapphire amplifier (Coherent, Astrella-F) was used to generate a 100 fs laser pulse train with a 1 kHz repetition rate and a central wavelength of 800 nm, which is split to generate both pump and probe. The pump wavelength is tuned by an optical parametric amplifier (Ultrafast Systems, Apollo-T), which yielded pump wavelengths of 505, 516, 560 and 600 nm to excite the B-exciton of both MoS_2 and WS_2 NPLs and NSs resonantly. The power was adjusted to low photoexcitation densities of 4.7 $\mu J/cm^2$, if no stated otherwise (excitation density dependent measurement series). The probe beam was variably delayed by a retro reflector to achieve the desired probe times after photoexcitation (up to 7.5 ns). For UV-vis/NIR spectra, the white light continuum (410-750 nm/880-1680 nm) was created by using sapphire crystals of differing thickness.

To allow for an accurate determination of decay times of partially convoluted signals, global analysis was performed. Both, MoS_2 and WS_2 NSs and NPLs were fitted by a sequential bi/tri-exponential decay convoluted with the instrument response function (IRF) using TAPAS, an open source software for TAS data editing and analysis.[1] The bi/tri-exponential model is derived and adapted from literature, where initial carrier-trapping and subsequent band gap renormalization are frequently discussed for colloidal TMDs.[2–5] A longer lived component was added to account for the residual bleach beyond the picosecond range, which is assumed to be connected to emissive recombination.

Chemicals

1,2-Dichlorobenzene (o-DCB, 99%), ethanol (99,8%), n-hexane (97%), methanol (99,9%), oleylamine (OlAm, 98%), oleic acid (OlAc, 90%) and sulfur (99.98%) were purchased from Sigma Aldrich. Molybdenum(V) chloride (99%) and tungsten(VI) chloride (99%) were purchased from Alfa Aesar and 1,1,1,3,3,3-hexamethyldisilazane (HMDS, 98%) was purchased from Acros Organics. Ascorbic acid (99%) was purchased from Thermo Scientific. OlAm was degassed under vacuum at 120 °C for 2 h before being stored under inert gas atmosphere inside a nitrogen filled glovebox. OlAc was degassed using the freeze–pump–thaw method and stored inside a nitrogen filled glovebox. $MoCl_5$, WCl_6, sulfur and HMDS are also stored inside a glovebox. All chemicals other than OlAm and OlAc were used as supplied without further purification.

Nanoplatelet (NPL) Synthesis

Metal precursor solution: In a nitrogen filled glovebox 2.2/3.0 mg (0.008 mmol) of $MoCl_5/WCl_6$ were dissolved in a mixture of OlAm (0.9 mL) and OlAc (0.1 mL).

Synthesis: 175 mg (5.46 mmol) of sulfur was dissolved in 34 mL of OlAm in a three-neck flask. The mixture was degassed in *vacuo* for 30 min. at 90 °C before being set under nitrogen. HMDS (0.5 mL, 2 mmol) was added and the solution was heated to 320 °C. Subsequently, the metal precursor was added dropwise over the course of 10 minutes. After the injection the reaction solution was stirred for another 2 h at 320 °C. Once the solution was cooled to room temperature, 70 mL of ethanol was

added and the mixture was centrifuged for 30 min at 4000 rpm. The supernatant was discarded, the NPLs were redispersed in 40 mL of hexane and centrifuged again for 10 min at 4000 rpm. After precipitation the hexane is discarded, concluding the washing step. Finally the washed NPLs were dissolved in o-DCB and stored under ambient conditions.

Nanosheet (NS) Synthesis

Metal precursor solution: In a glovebox 5.2/7.3 mg (0.019 mmol) of $MoCl_5/WCl_6$ were dissolved in OlAm (10 mL).

Synthesis: 8 mg (62.3 mmol) of sulfur was dissolved in 34 mL of OlAm within a three-neck flask. The mixture was degassed in *vacuo* for 30 min. at 90 °C before being set under nitrogen. HMDS (0.5 mL, 2 mmol) was added and the solution was heated to 320 °C. Subsequently, the metal precursor was added dropwise over the course of 1 h. After the injection the reaction solution was stirred for another 0.5 h at 320 °C. Once the solution was cooled to room temperature, 70 mL of ethanol was added and the mixture was centrifuged for 30 min at 4000 rpm. The supernatant was discarded, the NSs were redispersed in 40 mL of hexane and centrifuged again for 10 min at 4000 rpm. After precipitation the hexane is discarded, concluding the washing step. Finally the washed NSs were dissolved in o-DCB and stored under ambient conditions.

Ascorbic acid treatment

An excess of ascorbic acid (50 mg, 0.284 mmol) was dissolved in 0.5 mL methanol and added to a solution of WS_2 NPLs in 1 mL o-DCB. The solution was diluted with a 1:2 methanol/o-DCB mixture until 0.1 OD of optical density at the A-exciton is achieved.

Supplementary Experimental Data

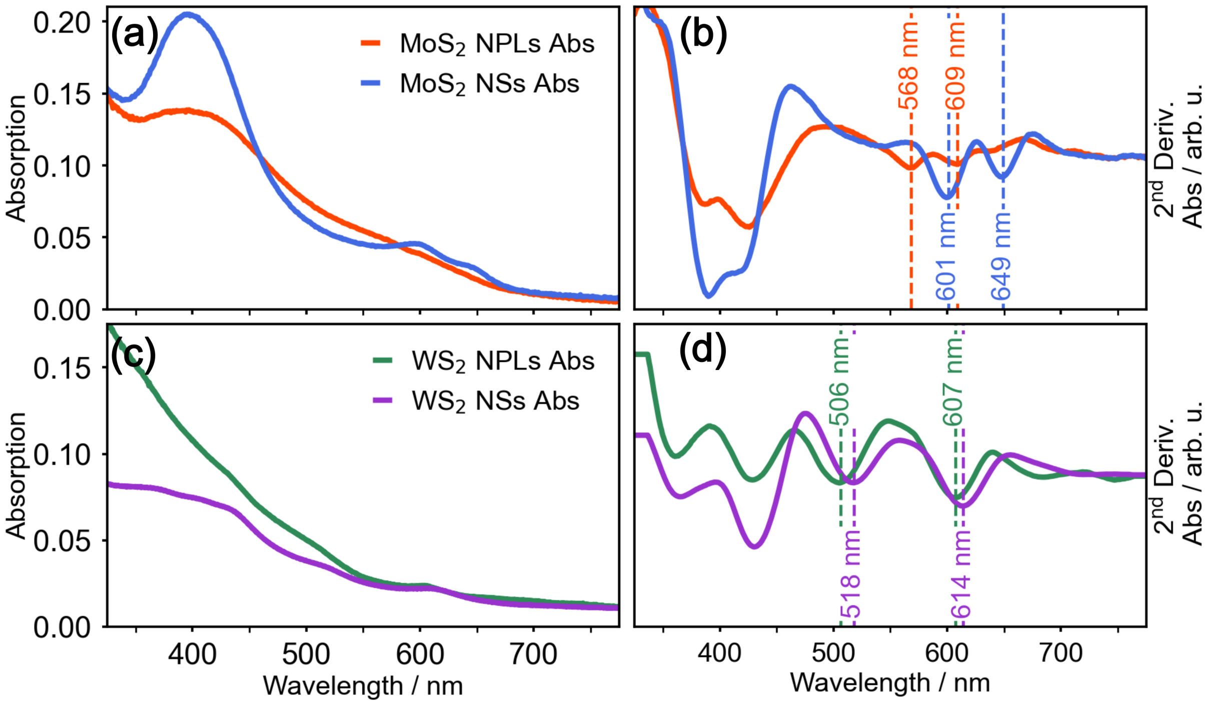

Figure S1: Steady- state UV-Vis absorption of MoS_2 (a) and WS_2 (c) NPLs/NS. Second derivative of MoS_2 (b) and WS_2 (d) absorption spectra for enhanced spectral resolution. A and B excitons can be distinguished, while a significantly lower A excitonic absorption is observed for MoS_2 NPLs compared to WS_2 NPLs.

Table S1: Spectral positions of excitonic in MoS_2 and WS_2 NPLs/NSs. Shifts of the A exciton upon increased lateral confinement in NPLs is calculated as difference of NSs and NPLs for each material.

	NSs	NPLs	Shift
MoS_2 A exciton	1.91 eV/649 nm	2.04 eV/609 nm	130 meV/40 nm
MoS_2 B exciton	2.06 eV/601 nm	2.18 eV/568 nm	
WS_2 A exciton	2.02 eV/614 nm	2.04 eV/607 nm	20 meV/7 nm
WS_2 B exciton	2.39 eV/518 nm	2.45 eV/506 nm	

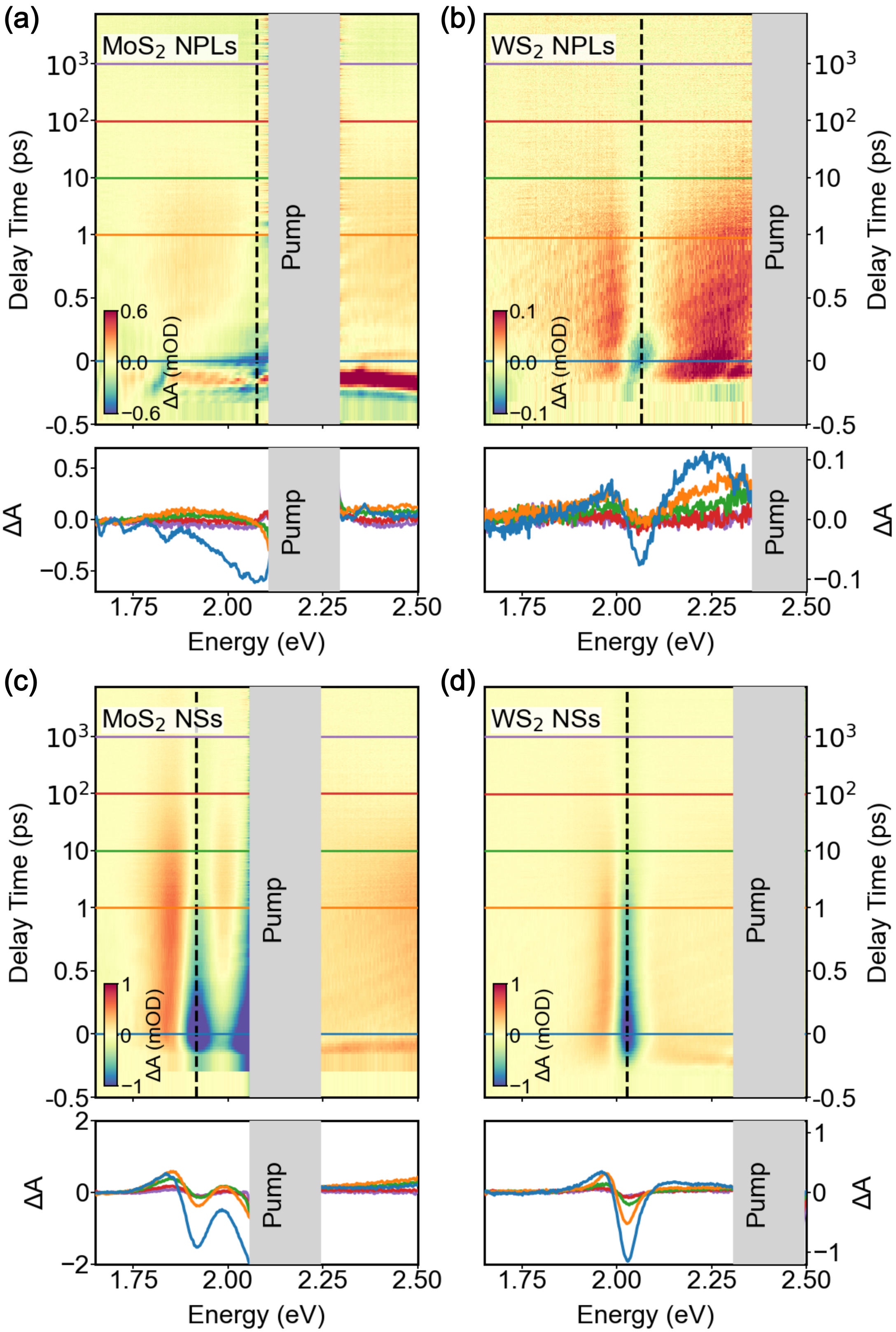

Figure S2: a-d) Hyperspectra and spectral line cuts taken at 0, 1, 10, 100 and 1000 ps (color of each line corresponds to the assigned spectral line cut) after photoexcitation of MoS_2 and WS_2 NS and NPL samples discussed in the main manuscript. Dashed line indicates where kinetic traces were acquired.

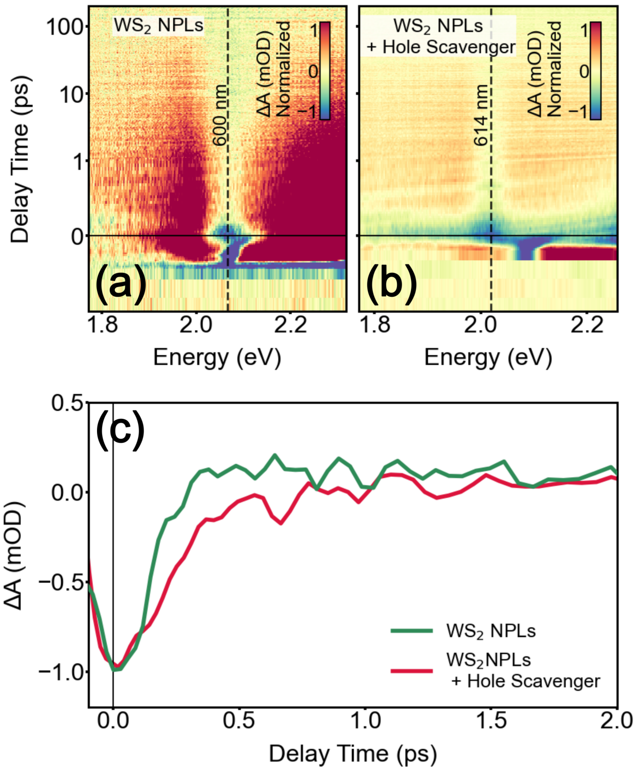

Figure S3: Hyperspectra of WS_2 NPLs (a) and WS_2 NPLs with ascorbic acid added as hole scavenger (b). (c) Decay traces taken along the dashed line in (a,b) show a longer initial trapping process when a hole scavenger is present.

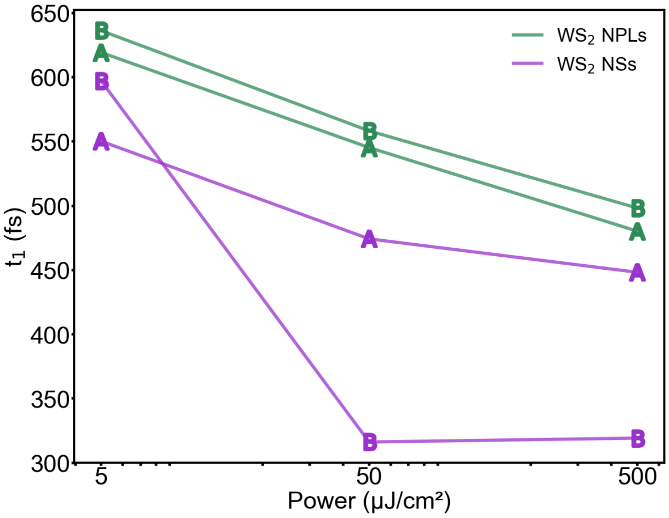

Figure S4: Photoexcitation density dependence of WS_2 NPLs and NSs, at the A and B excitonic transition. Resonant A excitation shows faster decay and implies a hole-mediated decay of the fast component in NPLs.

Computational Methods

The calculations were performed using density functional theory (DFT) as implemented in the Vienna Ab-initio Simulation Package (`VASP`).[6] The interactions between core and valence electrons were described using the projector augmented wave (PAW) method,[7] which accurately accounts for the all-electron character near atomic cores while maintaining computational efficiency. For the exchange–correlation functional, the generalized gradient approximation (GGA) in the Perdew-Burke-Ernzerhof (PBE)[8] formulation was used, which is widely adopted for semiconducting systems due to its reasonable balance between accuracy and computational cost. We used Grimme's D3 correction with Becke–Johnson (BJ) damping.[9,10] To simulate isolated nanoplatelets (NPLs) and prevent unphysical interactions between periodically repeated images in the simulation cell, a vacuum box of 16 Å was added in all directions around the NPLs to ensures that the simulated NPLs behave as non-periodic entities. A plane-wave energy cutoff of 475 eV was used to ensure convergence of the total energy and forces. Given the non-periodic nature of the NPLs, only the Gamma (Γ) point was used to sample the Brillouin zone, which is sufficient for large, isolated systems. The structures were fully relaxed with the total energy convergence criterion set to $1 \text{ x } 10^{-8}$ eV, and the atomic positions were optimized until the residual force on each atom was less than 0.005 eV/Å. We have also performed spin-polarized calculations and found these structures to be non-magnetic.

The orbital- and site-projected wavefunction characters are used to analyze the contribution of specific atomic orbitals and atomic sites to the electronic states. The analysis was carried out using in-house developed Python scripts, integrated with several open-source packages to extract and process data from `VASP` calculations. The `Pymatgen` library[11] was employed for structural analysis and the extraction of projected density of states (PDOS). The inverse participation ratio (IPR), which provides a quantitative measure of wavefunction localization, was computed using the `pyIPR` package.[12] To evaluate single-particle oscillator strengths, we used the `vasp_TDM` utility,[13] which calculates transition dipole moments in momentum representation from `VASP` outputs. For Bader charge analysis, the implementation developed by the Henkelman group was used.[14]

Supplementary Theory Results

In this section, we present additional results for the structures discussed in the main text. We first examine the effect of structural relaxation by evaluating the absolute atomic displacements from their initial positions, followed by an analysis of the nearest-neighbour distances for different atomic species. Finally, we provide the projected density of states (PDOS), resolved by element and orbital, for all the structures reported in the main text.

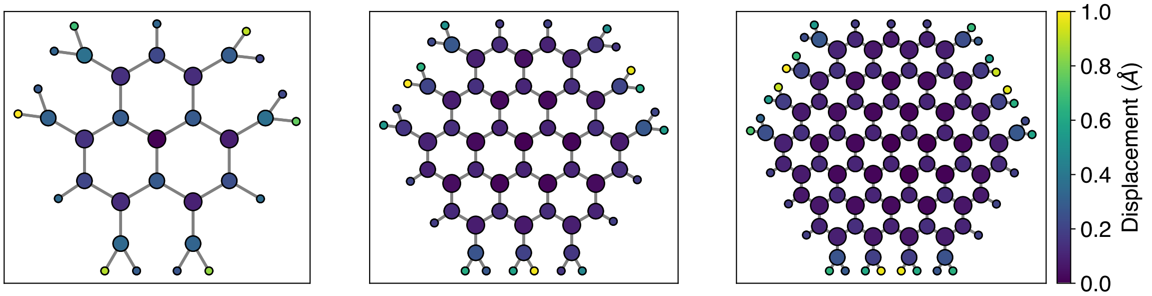

(a)

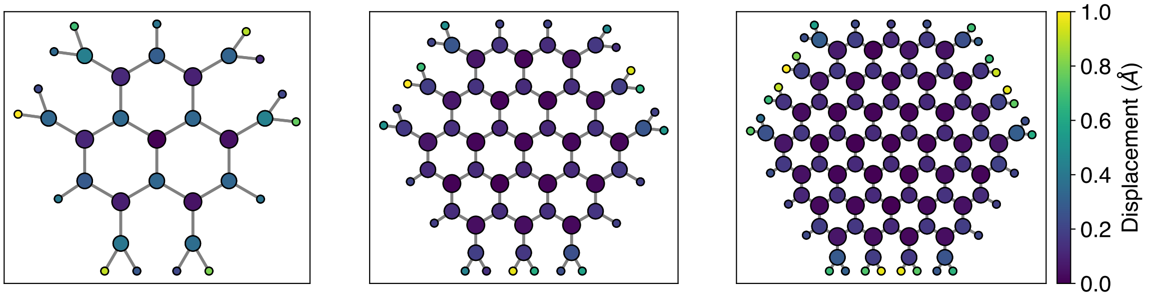

(b)

Figure S5: Absolute atomic displacement shown as a color scale on each atomic position after structural optimization of NPLs, with size increasing from left to right for (a) MoS_2 and (b) WS_2.

Atomic Position Displacement

Figure S5 presents the color-coded absolute atomic displacements for each atomic position after structural optimization, shown for NPLs of increasing size (left to right) for (S5a) MoS_2 and (S5b) WS_2. The results show that core atoms (both Mo/W and S) remain largely unchanged, whereas edge atoms undergo significant shifts, with H atoms displaying the largest displacements, highlighted in yellow. This behavior is expected, as the initial H positions were trial placements and thus experience stronger relaxation. As the size increases from 0.9 nm to 2.1 nm, the edge atoms shows

smaller relative displacements compared to those in the smallest structures, as expected. For a given size, the relaxation patterns are very similar for both materials.

Atomic Species Nearest Neighbour Distances

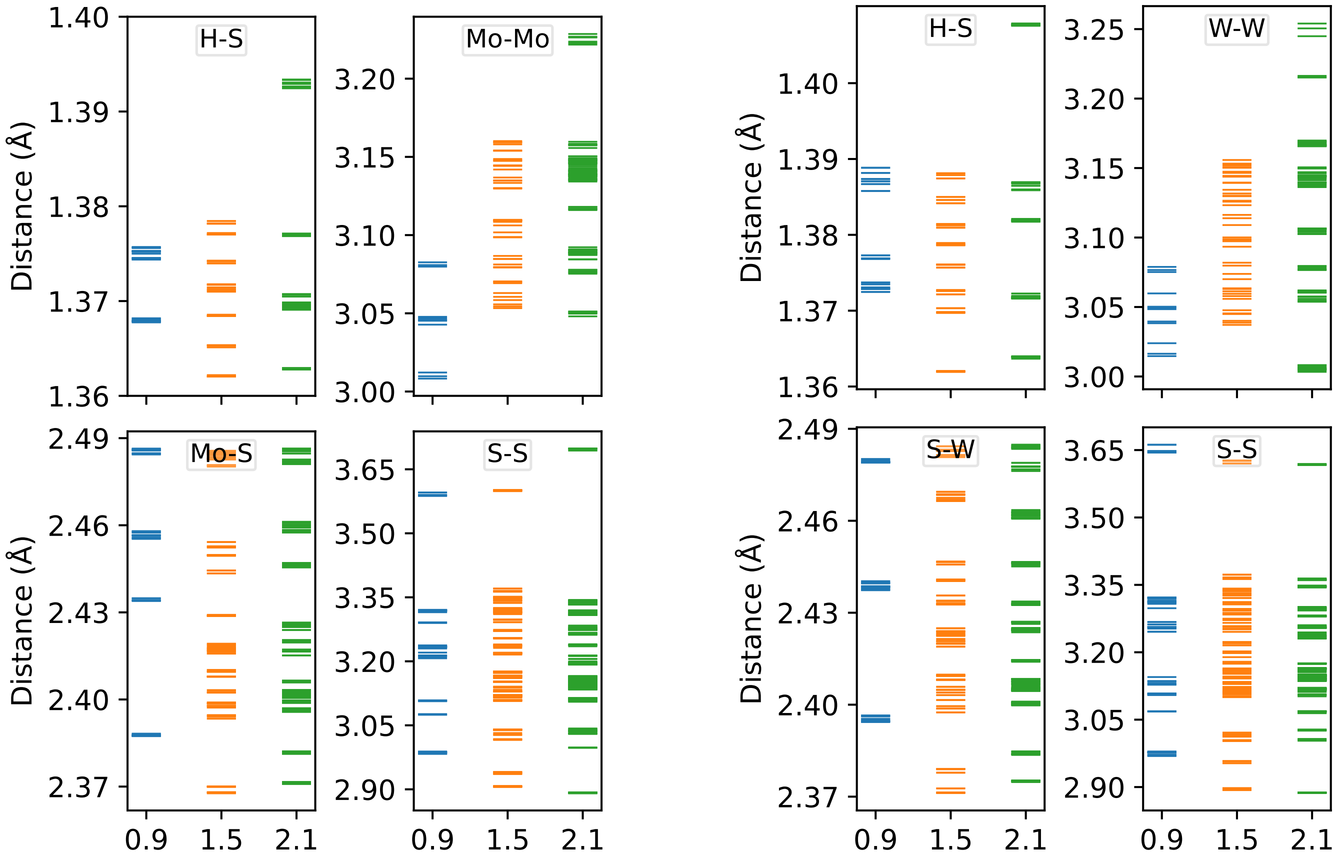

(a) (b)

Figure S6: First nearest-neighbor distances for each atomic species in NPLs, shown as a function of size after structural relaxation: (a) MoS_2 and (b) WS_2

In Figure S6, we present the atomic species nearest-neighbor distances after structural relaxation for all sizes of MoS_2 and WS_2 NPLs. These distances include Mo/W–S, S–S, Mo/W–Mo/W, and S–H bonds. For both materials, S–H bonds exhibit very little variation, with a small spread that slightly increases with NPLs size. The Mo/W–Mo/W distances also show relatively small variations and remain close to the experimental value of 3.13 Å, with WS_2 showing slightly longer distances and a marginally larger spread as the size increases. The Mo/W–S bond lengths are the most uniform, showing minimal spread across all sizes.

The largest variations are observed for S–S distances, which display a spread of up to 1 Å. This

is primarily due to surface S atoms, whose coordination differs significantly from core atoms because of the H-passivation at the edges. These atoms undergo stronger structural relaxation, as expected. This deviation is consistent with our Bader charge analysis, which shows that edge atoms carry different charges compared to core atoms. Changes in bond distances directly influence the charge distribution, providing a structural basis for the observed variations in edge versus core charges.

Electronic Density of States

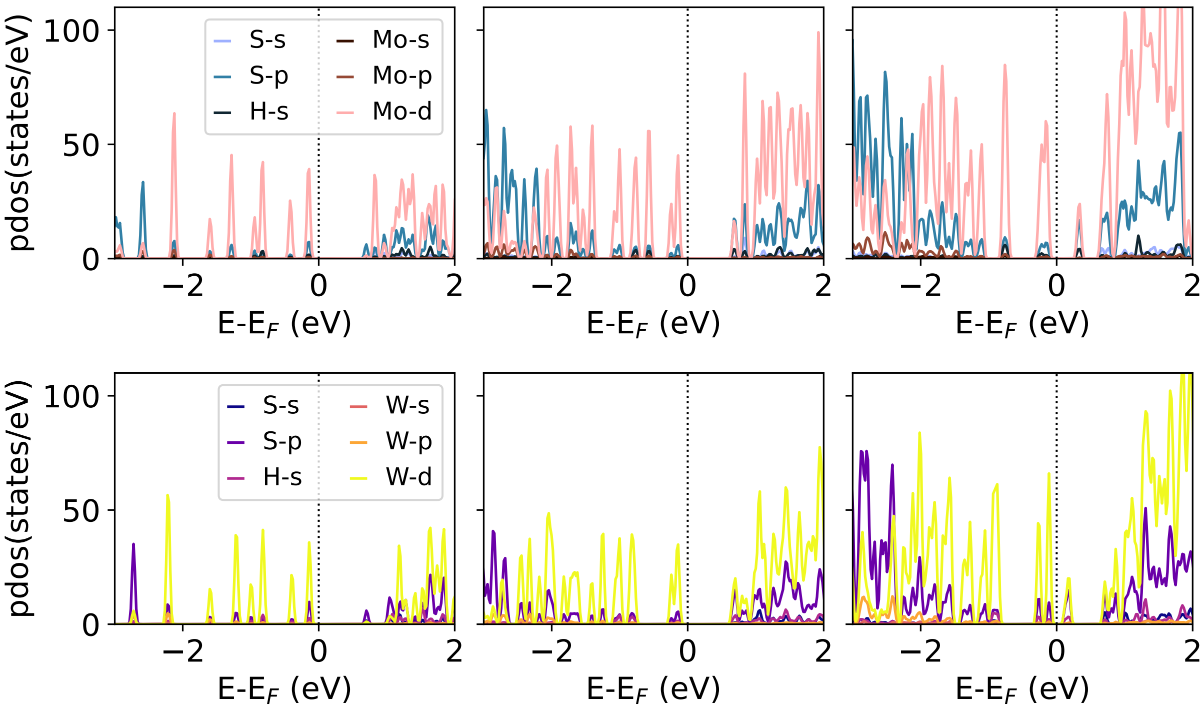

Figure S7: Projected density of states resolved by element and orbital for calculated structures from the main text, arranged by increasing size left to right: MoS_2 (top panel) and WS_2 (bottom panel).

In Figure S7, we show the element- and orbital-resolved projected density of states (PDOS) for all NPL sizes. Near the Fermi level (dotted black line), metal d orbitals dominate across all sizes and for both materials. S p-orbitals, the second-largest contributors, lie further from the Fermi level, while contributions from other orbitals are relatively minor. This clearly indicates that the metal atoms are the primary contributors to the trap states, as discussed in the main text. Another key characteristic observed in the PDOS of both materials is the shift from discrete spectral features in smaller NPLs toward broader, more continuous peaks with increasing size. This behavior unequivocally demonstrates the influence of lateral confinement.

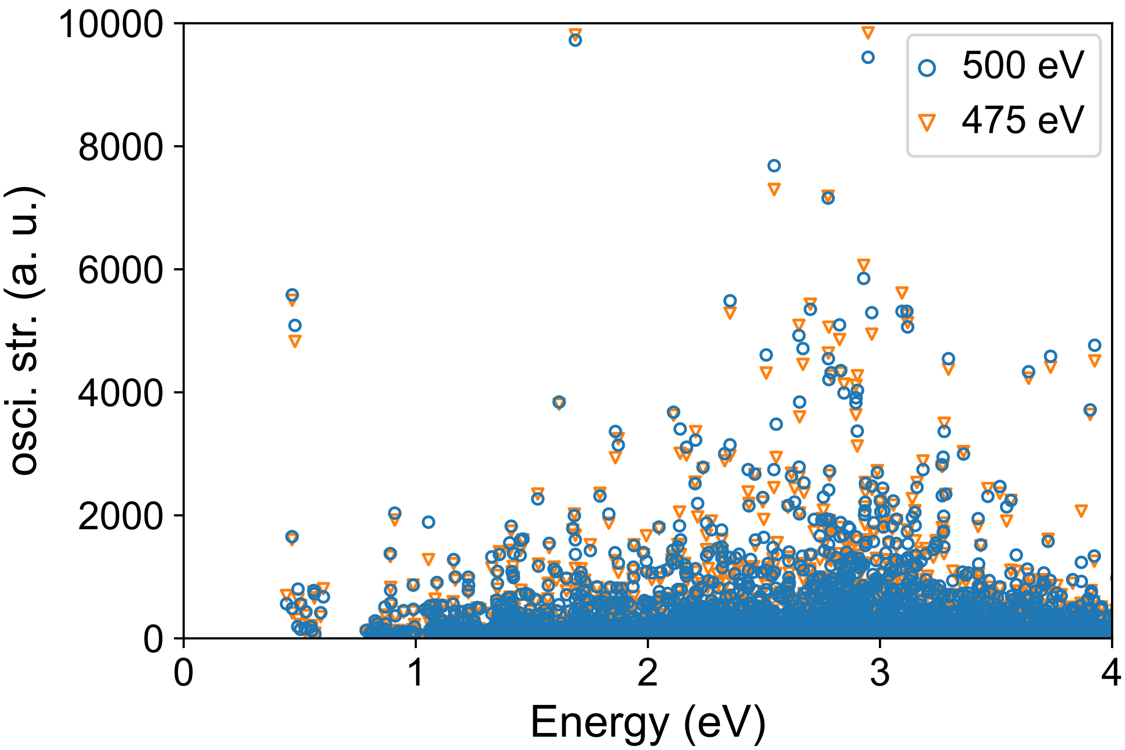

Figure S8: Comparison of oscillator strength for plane wave energy cutoff 475 and 500 eV, showing minor changes in intensities and spectral features.

References

(1) Frech, P.; Scheele, M. TAPAS: Transient Absorption Processing and Analysis Software. *ChemPhotoChem* **2026**, *10*, e202500236.

(2) Frauendorf, A. P.; Niebur, A.; Rudolph, D.; Oestreich, M.; Lauth, J.; Hübner, J. Ultrafast Recombination Dynamics under Lateral Confinement and Cryogenic Temperatures in Colloidal MoS_2. *J. Phys. Chem. C* **2024**, *128*, 16597–16606.

(3) Niebur, A.; Söll, A.; Haizmann, P.; Strolka, O.; Rudolph, D.; Tran, K.; Renz, F.; Frauendorf, A. P.; Hübner, J.; Peisert, H.; Scheele, M.; Lauth, J. Untangling the Intertwined: Metallic to Semiconducting Phase Transition of Colloidal MoS_2 Nanoplatelets and Nanosheets. *Nanoscale* **2023**, *15*, 5679–5688.

(4) Mutyala, C. S.; Pippia, G.; Tanghe, I.; Martín-García, B.; Rousaki, A.; Vandenabeele, P.; Schiettecatte, P.; Moreels, I.; Geiregat, P. Charge Carrier Dynamics in Colloidally Synthesized Monolayer MoX_2 Nanosheets. *J. Phys. Chem. Lett.* **2023**, *14*, 2620–2626.

(5) Schiettecatte, P.; Geiregat, P.; Hens, Z. Ultrafast Carrier Dynamics in Few-Layer Colloidal Molybdenum Disulfide Probed by Broadband Transient Absorption Spectroscopy. *J. Phys. Chem. C* **2019**, *123*, 10571–10577.

(6) Kresse, G.; Furthmüller, J. Efficient iiterative Schemes for AB Initio Total-energy Calculations using a Plane-wave Basis Set. *Phys. Rev. B* **1996**, *54*, 11169–11186.

(7) Kresse, G.; Joubert, D. From Ultrasoft Pseudopotentials to the Projector Augmented-wave Method. *Phys. Rev. B* **1999**, *59*, 1758–1775.

(8) Perdew, J. P.; Burke, K.; Ernzerhof, M. Generalized Gradient Approximation Made Simple. *Phys. Rev. Lett.* **1996**, *77*, 3865–3868.

(9) Grimme, S.; Antony, J.; Ehrlich, S.; Krieg, H. A Consistent and Accurate AB Initio Parametrization of Density Functional Dispersion Correction (DFT-D) for the 94 Elements H-Pu. *J. Chem. Phys.* **2010**, *132*, 154104.

(10) Grimme, S.; Ehrlich, S.; Goerigk, L. Effect of the Damping Function in Dispersion Corrected Density Functional Theory. *J. Comput. Chem.* **2011**, *32*, 1456–1465.

(11) Ong, S. P.; Richards, W. D.; Jain, A.; Hautier, G.; Kocher, M.; Cholia, S.; Gunter, D.; Chevrier, V. L.; Persson, K. A.; Ceder, G. Python Materials Genomics (pymatgen): A Robust, Open-source Python Library for Materials Analysis. *Comput. Mater. Sci.* **2013**, *68*, 314–319.

(12) Liu, H. pyIPR: A Python Tool to Calculate the Inverse Participation Ratio from VASP Outputs. `https://github.com/lhycms/pyIPR`.

(13) Zheng, Q. VASP_TDM: Transition Dipole Moment from VASP Outputs. `https://github.com/QijingZheng/VASP_TDM`.

(14) Tang, W.; Sanville, E.; Henkelman, G. Fast and Robust Algorithm for Bader Decomposition of Charge Density. *J. Phys.: Condens. Matter* **2009**, *21*, 084204.
